# Body dimension perception: a study among students of the military police officer training course

**DOI:** 10.3389/fpsyg.2026.1791464

**Published:** 2026-10-21

**Authors:** Roberta L. Rica, Eliane F. Gama, Geanderson S. Oliveira, Matheus F. de Deus, Valentina Bullo, Marco Bergamin, Stefano Gobbo, Danilo S. Bocalini

**Affiliations:** 1 Department of Physical Education, Estacio de Sá Universitary Center, Vitoria, Brazil; 2 Department of Morphology and Genetics, Federal University of São Paulo, São Paulo Medical School, São Paulo, Brazil; 3 Expimental and Biochemistry Laboratory, Physical Education Sport Center, Federal University of Espirito Santo, Vitoria, Brazil; 4 Department of Medicine, University of Padova, Padova, Italy; 5 Gymhub S.r.l. Spin-off of the University of Padova, Padova, Italy

**Keywords:** body, body perception, military police, police, soldiers

## Abstract

**Introduction:**

Analyzing body size perception among Military Police Officers is important for their work. Thus, the aim of the study was to analyze body dimension perception among students of the Military Police Officer Training Course (MPOC).

**Methods:**

MPOC students were evaluated for body dimension perception and variables such as anthropometric measures and body composition, mood state, and physical activity time.

**Results:**

In this study, we found that 66% of MPOC students had adequate body size perception, 26% underestimated body size and 8% overestimated. No correlations were observed between body perception index indicators and anthropometric parameters, physical activity time, or mood.

**Conclusion:**

In summary, body dimension perception among future officers is not merely an evaluation of one’s own body but reflects the complex interaction between physical reality and the pressures of the military context.

## Introduction

Public security is considered a topic of intense debate and faces complex challenges, requiring security agents to be physically and mentally prepared to act in situations of high stress and risk. Given the reality faced by public security professionals, military police officers deal daily with high-risk situations, including constant exposure to violence, the imminent threat of death, long working hours, and adverse working conditions. These factors, combined with the stress inherent to police work, contribute to making them one of the groups most susceptible to work-related health problems (Marinho et al., 2018; Souza and Minayo, 2005).

Thus, the military police profession is among the most demanding both physically and emotionally, requiring not only technical and tactical skills but also a high level of physical fitness, emotional control, and perceptual abilities involving both the environment and the body itself (Marinho et al., 2018; Souza and Minayo, 2005). Consequently, police response capacity in armed confrontations and high-tension situations is directly related to aspects such as muscle strength, balance, breathing control, anxiety, and motor efficiency (Marinho et al., 2018; Souza and Minayo, 2005). In this context, physical training and psychological preparation are fundamental to ensuring adequate performance in real conflict situations. However, there is a gap in the scientific literature regarding information on body dimension perception among military police officers.

Body dimension perception, a multifaceted construct, encompasses the attitudinal domain related to feelings and thoughts about the body and the dimensional domain, which refers to the neurological and spatial representation of one’s own body. The latter, in particular, functions as a fundamental guide for motor planning (Ramsey and Van Der Meer, 2022). As argued by leading authors in the field, such as Nicholas Holmes, the ability to accurately estimate the dimensions of one’s own body and its relation to the environment is essential for the execution of complex movements and for interaction with objects and spaces, being one of the pillars of body representation (Holmes and Spence, 2004; Cardinali and Holmes, 2023).

However, the accuracy of this perception is not uniform and manifests differently between genders. Recent literature has consistently demonstrated that women tend to overestimate their body size (Papadopoulos, 2020; Mishra and Dasgupta, 2021), whereas men commonly underestimate it (Vartanian, 2020; Melchior, 2022). The clinical and occupational relevance of this disparity becomes evident when considering professions that require sharp dimensional perception, such as Military Police officers. The question that arises is whether body-size underestimation, commonly observed among men, may have direct implications for professionals who, in their work activities, need precise spatial assessment to ensure their own safety and that of others.

Given the above and the gap identified in the literature, the present study aims to analyze body dimension perception among students of the Military Police Officer Training Course, investigating the relationship between this perception and variables such as body composition measures, mood state, and physical activity time.

## Materials and methods

### General characteristics and sample selection

After approval from the Research Ethics Committee in Humans of the Federal University of Espírito Santo and authorization from the Directorate of Education of the Military Police of Espírito Santo (DMPES), military police officers of both sexes from the Officer Training Course of the Military Police of Espírito Santo (MPES) were invited to voluntarily participate in the study. The participants, after consenting, signed a Free and Informed Consent Form, in accordance with the norms established in Resolution No. 466/2012 of the National Health Council.

The invitation to participate in the study was made through direct contact with the military personnel and by verbal and digital dissemination strategies. The inclusion criteria were as follows: active duty military police officers, a minimum of 1 year of operational service. As non-inclusion, military police officers on leave from work for any reason during the study period, as well as the non-signing of the free and informed consent form, were used. The exclusion criteria included officers who were temporarily removed from duty due to medical or psychological conditions, those who did not sign the informed consent form, and those who did not complete all study procedures.

Military personnel who did not respond to or participate in all collections and analyses of the study were excluded. After applying the inclusion, non-inclusion, and exclusion criteria, 15 military (4 female and 11 male) police officers were from the Officer Training Course of MPES.

### Data collection procedure

The Officer Training Course of MPES is the initial and essential stage for entering the career of a combatant officer, aimed at preparing leaders and managers in public security. Held at the Military Police Academy of Espírito Santo (MPAES), on a full-time basis and lasting 3 years, the Officer Training Course is a bachelor’s degree course in Police Sciences and Public Security that combines technical, legal, operational, and managerial training. The course workload extends over three academic years and is delivered at MPAES, with curricular components covering the core operational, legal, management, and ethics domains. During the course, military police officers face an intense routine of studies, physical and practical training, supervised internships, and participation in institutional activities, focusing on the development of competencies such as leadership, decision-making under pressure, discipline, ethics and involves an average of 3 years of preparation.

The data collection stage took place on the premises of the MPAES, during working days and according to the availability of the participants in the last month of the course. Initially, the volunteers underwent anthropometric measurements and handgrip strength tests, wearing the specific uniform for military physical training. Subsequently, they answered self-report instruments that addressed aspects related to perceived stress levels and habitual physical activity practice.

### Evaluated parameters

#### Body dimension perception

Body dimension perception was assessed using the Image Marking Procedure, as previously described by Rica and Gama (2018a). Briefly, tactile assessment was conducted at predetermined points. Participants were marked with tape on the right and left acromioclavicular joints, right and left waist curves, and right and left greater trochanters using colored adhesive labels (green, blue, red, and black) to ensure consistency across three trials.

Participants stood near a wall where a vertical measuring tape had been fixed as a reference. The researcher touched each marked point on the participant’s body, and the participant indicated the orthogonal projection of that point on the wall, which was then marked with a colored label. The sequence of points was: top of the head, right shoulder, waist, hip, then left shoulder, waist, and hip. The test was repeated three times, and participants could not see previous markings.

After perceived measures were collected, the examiner positioned the blindfolded participant near the wall to maintain proper foot alignment. Real body point heights were then measured using the measuring tape. Labels and the wall tape were photographed for later analysis using AxioVision Version 3.1.

Quantitative analyses were performed by calculating distances between perceived and actual points, evaluating both height and width in the horizontal plane. Data were tabulated, and the body perception index (BPI) was calculated as the ratio between perceived and actual measurements multiplied by 100. The total BPI was computed as the average of regional BPIs (Rica and Gama, 2018a; Rica and Gama, 2018b). Values above 112.3% indicated hyper-schematism, whereas values below 99.4% indicated hypo-schematism and adequate values around 99.4–112.3%.

#### Anthropometric measures and body composition

For anthropometric evaluation, body mass (MarteScientific LS200P digital scale; precision 0.1 kg) and height (Cardiomed WCS stadiometer; precision 0.1 cm) were measured. Body mass index (BMI) was calculated as body mass divided by squared height. Nutritional status was classified based on BMI (kg/m^2^): Normal (>18.5, <25.0), Overweight (≥25.0, <30.0), and Obese (≥30.0, <40.0).

Body composition was estimated using a doubly indirect method through measurements of bicipital, tricipital, subscapular, and suprailiac skinfolds (Mitutoyo/Cescorf caliper, precision 0.1 mm). Body fat percentage (%BF) was estimated according to Durnin and Womersley. %BF was classified as: normal (<18%), overweight (18–24.9%), and obese (>25%), according to Sampaio et al. (2024).

Waist circumference (WC) was measured with an elastic tape at the midpoint between the lower edge of the last rib and the upper edge of the iliac crest. Cardiovascular risk was classified as: increased risk (men ≥ 94 cm; women ≥ 80 cm) and substantially increased risk (men ≥ 102 cm; women ≥ 88 cm), following Sampaio et al. (2024).

### Mood state

Mood was assessed using the Brunel Mood Scale (BRUMS), derived from the Profile of Mood States (POMS), in concordance with previous studies (Allocca Filho et al., 2022). Mood is recognized as an important marker of transient emotional state, directly affecting concentration, fine motor control, risk perception, and reaction time, skills essential for the precise and safe execution of firearm use. Variations in subscales such as fatigue, tension, confusion, or anger can compromise self-control and motor stability, consequently impairing shooting accuracy.

The BRUMS questionnaire evaluates six affective dimensions: tension, depression, anger, vigor, fatigue, and confusion. The negative mood factors (tension, depression, anger, fatigue, confusion) are contrasted with the positive factor (vigor). The total mood disturbance (TMD) was calculated as the sum of negative factors minus the vigor score (TMD = [T + D + A + F + C] – V), with an additional 100 points added to avoid negative totals. Participants were instructed to respond according to how they were feeling “right now, at this very moment”.

### Physical activity time

Total weekly physical activity (PA) time was utilized to classify the PA level according to the International Physical Activity Questionnaire (IPAQ-short version), as applied in other studies (Vasconcelos Junior et al., 2025; Oliveira et al., 2023; Manzolli et al., 2024; Dutra et al., 2023). The questionnaire explored the frequency and duration of physical activities, including walking and moderate and vigorous exercise. All subjects were considered active or meeting physical activity (PA) recommendations if they achieved 150 min or more PA per week; those who did not meet this threshold were classified as inactive.

### Statistical analysis

Data are presented as absolute (n) and relative (%) frequency for qualitative variables, and as mean ± standard deviation, coefficient of variation (CV), and 95% confidence interval (95% CI) for quantitative variables. Pearson correlation tests were used to determine the strength and direction of correlations between body dimension perception parameters, physical activity time, and mood. Correlation strength classification: 0.10 to 0.30 (weak), 0.40 to 0.60 (moderate) and 0.70 to 1.00 (strong). Analyses were performed using GraphPad Prism version 6.00 for Windows (GraphPad Software, La Jolla, California, United States), with significance level set at *p* < 0.05.

## Results

The age of the military students was 30.87 ± 2.29 years (CV: 7.43%), with a service time of 13.93 ± 5.00 years (CV: 35.93%). Anthropometric parameters are presented in Table 1. Overall, the sample showed an elevated BMI; however, body fat percentage and cardiovascular risk assessed by waist circumference were both low.

**Table 1:** General characteristics of students enrolled in the MPES officer training course.

Parameters	Mean ± SD	CV (%)	95% CI
Age (years)	30.87 ± 2.29	7.43	29.60–32.14
Service time (years)	13.93 ± 5.00	35.93	11.16–16.71
Body mass (kg)	80.45 ± 6.35	7.89	76.93–83.97
Height (m)	1.76 ± 0.05	3.24	1.73–1.79
BMI (kg/m2)	25.93 ± 2.01	7.75	24.81–27.04
% body fat	12.29 ± 3.88	31.57	10.14–14.44
Fat mass (kg)	9.89 ± 3.16	32.00	8.14–11.65
Fat free mass (kg)	70.55 ± 6.19	8.78	67.12–73.98
Muscle mass (kg)	39.67 ± 3.68	9.28	37.63–41.71
Waist circumference (cm)	83.67 ± 4.29	5.12	81.29–86.04

Values expressed as mean ± standard deviation, coefficient of variation (CV), and 95% confidence interval (95% CI).

Mood-related parameters and physical activity levels can be found in Table 2. It is important to note that the average weekly physical activity time across all domains was considered high, exceeding the physical activity recommendations of the World Health Organization.

**Table 2:** Mood and physical activity of students enrolled in the MPES officer training course.

Parameters	Mean ± SD	CV (%)	95% CI
Mood state			
Tension	2.60 ± 1.50	57.78	1.76–3.43
Depression	1.26 ± 1.38	109.50	0.49–2.03
Anger	2.86 ± 3.98	138.80	0.66–5.07
Vigor	5.86 ± 3.15	53.85	4.11–7.61
Fatigue	5.40 ± 3.29	60.94	3.57–7.22
Mental confusion	0.73 ± 1.16	158.60	0.08–1.37
Mood disturbance	107.00 ± 9.62	8.99	101.70–112.30
Physical activity time			
Light (min)	243.30 ± 347.20	142.70	51.05–435.60
Moderate (min)	154.30 ± 159.20	103.10	66.19–242.50
Intense (min)	188.70 ± 243.30	124.20	58.91–318.40
Total (min)	586.30 ± 662.80	113.00	219.30–953.40

Values expressed as mean ± standard deviation, coefficient of variation (CV), and 95% confidence interval (95% CI).

Regarding body dimension perception, as shown in Table 3, values ranged from the lowest BPI for the shoulder segment (77.70 ± 13.88; CV: 17.86%) to the highest BPI for the waist segment (92.86 ± 21.36; CV: 23.00%). Considering the overall BPI, most police officers demonstrated adequate body perception (*n* = 10; 66%), followed by underestimation of body size (*n* = 4; 26%) and overestimation (*n* = 1; 8%).

**Table 3:** Body perception index of students enrolled in the MPES officer training course.

Parameters	Mean ± SD	CV (%)	95% CI
Head	92.58 ± 5.04	5.44	89.78–95.37
Shoulder	77.70 ± 13.88	17.86	70.01–85.38
Waist	92.86 ± 21.36	23.00	81.04–104.70
Hip	91.78 ± 25.64	27.94	77.58–106.00
General	88.73 ± 14.77	16.65	80.55–96.91

Values expressed as mean ± standard deviation, coefficient of variation (CV), and 95% confidence interval (95% CI).

This statement refers to our statistical findings indicating that Pearson correlation tests revealed no significant relationship (*p* > 0.05) between the Body Perception Index (BPI), which quantifies the accuracy of body dimension estimation, and the other studied variables, including anthropometric parameters (BMI, body fat percentage), physical activity time, or mood states (Table 4).

**Table 4:** Correlations between body perception index (BPI) and anthropometric indicators, physical activity time, and mood of MPES officer training course.

Parameters	BPI head	BPI shoulder	BPI waist	BPI hip	BPI general
Antropométrics					
Body mass (kg)	0.721	0.431	0.431	0.688	0.486
Height (m)	0.480	0.980	0.980	0.532	0.711
BMI (kg/m2)	0.818	0.407	0.407	0.306	0.275
% body fat	0.074	0.304	0.304	0.167	0.223
Fat mass (kg)	0.159	0.257	0.257	0.149	0.210
Fat free mass (kg)	0.173	0.218	0.218	0.157	0.188
Muscle mass (kg)	0.290	0.851	0.851	0.775	0.954
Waist circumference (cm)	0.111	0.844	0.844	0.768	0.944
Mood state					
Tension	0.636	0.172	0.199	0.306	0.202
Depression	0.131	0.794	0.666	0.273	0.491
Ange	0.151	0.824	0.858	0.709	0.770
Vigor	0.263	0.427	0.741	0.435	0.716
Fatigue	0.682	0.979	0.758	0.505	0.711
Mental confusion	0.491	0.918	0.971	0.867	0.886
Mood disturbance	0.236	0.873	0.588	0.326	0.496
Physical activity time					
Light	0.527	0.680	0.824	0.840	0.971
Moderate	0.760	0.709	0.910	0.963	0.934
Intense	0.588	0.419	0.458	0.915	0.584
Total	0.832	0.554	0.686	0.937	0.817

## Discussion

Among the main points highlighted in this study, it is important to note that the evaluated participants were students of the Military Police Officer Training Course, individuals who are regularly subjected to high demands and routine physical training, as well as robust psychological preparation, particularly due to their professional role as public security managers.

It is important to emphasize that professional routines may cause physical and emotional strain on workers, resulting in health impairments and affecting their perception of quality of life (Souza Filho et al., 2015). Benevides-Pereira (2002) considers military work one of the professions most exposed to risks, with notable threats to health and integrity. A relevant point concerns the study population, to our knowledge, only one study has evaluated military personnel enrolled in the Military Police Officer Training Course (Vasconcelos Junior et al., 2025), and the predominance of male participants in the present sample is consistent with the demographic profile observed in other studies conducted by our research group (Vasconcelos Junior et al., 2025; Manzolli et al., 2024; Oliveira et al., 2023; Dutra et al., 2023). According to Lima et al. (2015), male predominance may be related to the recruitment process of the Military Police, since the number of vacancies for women in public admission notices is usually lower. However, Vasconcelos Junior et al. (2025) highlight that admission notices for the MPES Officer Training Academy do not differentiate or limit vacancies based on gender, preventing the attribution of male predominance to administrative practices.

Regarding anthropometric parameters, although our results showed overweight according to the mean BMI (25.93 ± 2.01 kg/m^2^), the body fat (12.29 ± 3.88%) was considered adequate (Table 1). High body mass prevalence among military police officers has been documented in several studies (Oliveira et al., 2023; Manzolli et al., 2024; Santos M. M. et al., 2021; Santos H. M. et al., 2021; Itacarambi et al., 2019); however, our findings were similar to those of Vasconcelos Junior et al. (2025). Although overweight prevalence in military populations is common, we suggest that evaluating obesity in military personnel should not rely solely on BMI. Other parameters, such as body fat percentage, should also be used to obtain a more consistent assessment.

Similarly to Vasconcelos Junior et al. (2025), the low body fat percentage observed in the present study may be associated with the high physical activity demands imposed on cadets (Boçon, 2015), as presented in Table 2. Studies conducted by our research group have consistently demonstrated that military police officers from Espírito Santo exhibit high levels of physical activity (Vasconcelos Junior et al., 2025; Oliveira et al., 2023; Manzolli et al., 2024; Dutra et al., 2023), which may be related to the socioeconomic, environmental, and lifestyle characteristics of the region (IBGE, 2023; Manzolli et al., 2024; Oliveira et al., 2023; Dutra et al., 2023; Rosário et al., 2022; Agrizzi et al., 2021; Prando et al., 2012).

Regarding mood evaluation, it is noteworthy that the BRUMS instrument is frequently used in sports contexts (Rotta et al., 2014; Rohlfs et al., 2008) but is scarcely explored among public security personnel (Andrade et al., 2016). Mood state can be defined as an emotional condition that varies in intensity and duration and normally involves more than one factor (Lane and Terry, 2000), including negative states such as tension, depression, anger, fatigue, and confusion, and a positive state, vigor. Mood states may reflect feelings of happiness, excitement, sadness, distress, among others. A mood profile considered ideal for optimal sports performance is characterized by elevated vigor compared to negative mood factors, known as the Iceberg Profile (Morgan, 1980).

According to Werneck et al. (2006), changes in mood may influence decision-making and motor skill performance, impairing cognitive functioning. Thus, some studies (Dominski et al., 2015; Strahler and Ziegert, 2015; Bolmont et al., 2000) have shown that motivation, stress, anxiety, and anger can affect police performance. Moreover, police fatigue increases the likelihood of alterations in attention, concentration, and memory, and is also associated with reduced psychomotor capacity (Strahler and Ziegert, 2015).

In our study, negative mood indicators were considered low (Table 2); however, fatigue levels were similar to vigor, compromising the Iceberg Profile. Vigor, a positive mood dimension, is characterized by physical energy, enthusiasm, and motivation, and is inversely related to fatigue and depression (Terry, 1995; Rohlfs et al., 2008). Considering findings from Vasconcelos Junior et al. (2025), which revealed that 45.8% of military personnel exhibited symptoms of stress, we believe that course activities such as final project presentation, operational internships, and academic evaluations may have increased fatigue, as demonstrated by Sousa et al. (2020).

Regarding body dimension perception, the results showed that although most participants demonstrated adequate perception, the group tended to underestimate their own body size. This finding is reinforced by the overall average of 88.73% (± 14.77%), below the minimum threshold of 99.4% considered adequate (Segheto et al., 2011), and by the predominance of underestimation as the second most common perception pattern.

Segment-specific analysis revealed notable discrepancies. Waist perception, closest to the ideal value (92.86%), suggests greater accuracy in perceiving this region. In contrast, shoulder perception had the lowest average (77.70%), indicating substantial underestimation. In studies on male body perception, waist-to-hip ratio and shoulder-to-waist ratio are often analyzed as indicators of attractiveness and strength (Thompson et al., 1999). Shoulder width is frequently associated with masculinity and strength; thus, underestimation in a physically conditioned group suggests that the “ideal” male body, symbolized by broad shoulders, may be so inflated that cadets, even those with well-developed physiques, do not perceive themselves as sufficiently wide. This discrepancy between perceived dimension and masculine body ideals may contribute to dissatisfaction, even among individuals nearing the physical ideal.

The accurate perception of waist width contrasted with underestimation of shoulder width may reflect expectations and bodily ideals within the military context. A slimmer waist is an indicator of good physical conditioning, highly valued and measurable through training routines, leading to a more realistic and less distorted perception. In contrast, shoulder width may be influenced by aesthetic and symbolic expectations linked to military identity. As Campana et al. (2014) highlight, muscular strength and a robust physique are not merely aesthetic ideals in the military context but may be perceived as prerequisites for professional success. Our previous research on body perception among special operations military police officers in Vitória indicated that uniforms influence body perception, and that these officers tend to perceive themselves as smaller when not wearing uniforms [Universidade Federal do Espírito Santo (UFES), 2024]. Although shoulder and waist perception were not specifically analyzed in that study, it supports the idea that military environments and attire directly influence perception of body dimensions.

The absence of correlations between body perception indicators and variables such as anthropometric parameters, physical activity time, and mood is one of the most intriguing findings. Contrary to literature that often associates body perception with physical and emotional characteristics, such as body dissatisfaction influencing mood (Cash, 2002), our results suggest that body perception among participants is not directly influenced by their physical measurements, exercise habits, or emotional state. This unique finding may be explained by the rigorous and standardized environment of the training course. Thompson et al. (1999) note that sociocultural factors shape body perception, and in military settings, pressure to fit an “ideal” physique, strict discipline, and training-related stress may override individual perceptions, making body perception more reflective of group expectations than personal characteristics.

This study investigated body dimension perception among Military Police Officer trainees and revealed that although most participants exhibited adequate perception, the group tended to underestimate their own body size. The most notable discrepancy was the accurate perception of waist width contrasted with underestimation of shoulder width.

The most relevant finding was the absence of correlations between body perception and factors such as anthropometric measures, physical activity levels, and mood.

Some limitations of the present study must be acknowledged, particularly with respect to the potential for erroneous generalizations. These limitations include the relatively small sample size and the predominance of male participants; the assessment of physical activity using self-administered questionnaires, which may have introduced substantial variability in the data; the focus on military personnel not primarily engaged in operational duties. New studies looking at the relationship between body dimension perception and performance outcomes relating to tactical planning and movement in confined spaces, could help clarify its influence.

In summary, body dimension perception among officers may reflect the complex interaction between physical reality and the pressures of the military context. Understanding this dynamic is essential for improving the training of these professionals, ensuring that body dimension perception aligns with the demands of the profession, for both safety and effectiveness.

## Data Availability

The raw data supporting the conclusions of this article will be made available by the authors under reasonable request.
